# TReating Incontinence for Underlying Mental and Physical Health (TRIUMPH): a study protocol for a multicenter, double-blinded, randomized, 3-arm trial to evaluate the multisystem effects of pharmacologic treatment strategies for urgency-predominant urinary incontinence in ambulatory older women

**DOI:** 10.1186/s13063-023-07279-z

**Published:** 2023-04-21

**Authors:** Alison J. Huang, Louise C. Walter, Kristine Yaffe, Eric Vittinghoff, Erica Kornblith, Michael Schembri, Ann Chang, Leslee L. Subak

**Affiliations:** 1 University of California San Francisco, San Francisco, USA; 2 San Francisco Veterans Affairs, San Francisco, USA; 3 Stanford University, Stanford, USA

**Keywords:** Urinary urge incontinence, Cognitive dysfunction, Antimuscarinic agents, Adrenergic beta-3 receptor agonists

## Abstract

**Background:**

Urgency-type urinary incontinence affects one in four older community-dwelling women and overlaps with other common aging-associated health syndromes such as cognitive impairment, physical mobility impairment, and depression. Observational studies have raised concern about potentially higher rates of delirium and dementia in older adults taking anticholinergic bladder medications, but few prospective data are available to evaluate the effects of these and other pharmacologic treatments for urgency incontinence on cognition and other multisystem functional domains important to older women.

**Methods:**

The TRIUMPH study is a randomized, double-blinded, 3-arm, parallel-group trial comparing the multisystem effects of anticholinergic versus beta-3-adrenergic agonist bladder therapy and versus no active bladder anti-spasmodic pharmacotherapy in older women with urgency incontinence. Women aged 60 years and older (target *N* = 270) who have chronic urgency-predominant urinary incontinence and either normal or mildly impaired cognition at baseline are recruited from the community by investigators based in northern California, USA. Participants are randomized in equal ratios to take identically encapsulated oral anticholinergic bladder therapy (in the form of tolterodine 2 mg extended release [ER]), oral beta-3 adrenergic agonist bladder therapy (mirabegron 25 mg ER), or placebo daily for 24 weeks, with the option of participant-directed dose titration (to tolterodine 4 mg ER, mirabegron 50 mg ER, or matching placebo daily). Participants also receive patient-oriented information and instructions about practicing first-line behavioral management strategies for incontinence. The primary outcome is change in composite cognitive function over 24 weeks assessed by a comprehensive battery of cognitive tests, with a secondary exploration of the persistence of change at 36 weeks. Secondary outcomes include changes over 24 and 36 weeks in domain-specific cognitive function; frequency, severity, and impact of urgency-associated urinary symptoms; physical function and balance; sleep quality and daytime sleepiness; psychological function; and bowel function.

**Discussion:**

The TRIUMPH trial addresses the need for rigorous evidence to guide counseling and decision-making for older women who are weighing the potential multisystem benefits and risks of pharmacologic treatments for urgency incontinence in order to preserve their day-to-day functioning, quality of life, and independence in older age.

**Trial registration:**

ClinicalTrials.gov NCT05362292. Registered on May 5, 2022.

## Administrative information

**Table:** 

Title {1}	TReating Incontinence for Underlying Mental and Physical Health (TRIUMPH): a study protocol for a multicenter, three-arm, double-blinded randomized trial to evaluate the multisystem effects of pharmacologic treatment strategies for urgency-predominant urinary incontinence in ambulatory older women
Trial registration {2a and 2b}	ClinicalTrials.gov Identifier: NCT05362292. All items from the WHO Trial Registration Dataset can be found in this protocol or in the trial’s clinicaltrials.gov listing
Protocol version {3}	Protocol version 1.5, as of December 8, 2022
Funding {4}	National Institute on Aging grants R01AG075471 and K24AG068601 (no other financial, material, or other support)
Author details {5a}	Authors: Alison J. Huang, MD, MAS^1^; Louise C. Walter, MD^1^; Kristine Yaffe, MD^1^; Eric Vittinghoff, PhD^1^; Erica Kornblith, PhD^1,2^; Michael Schembri, BS^1^; Arunee Ann Chang, BS^1^; Leslee L. Subak, MD^3^ Affiliations: ^1^University of California San Francisco; ^2^San Francisco Veterans Affairs; ^3^Stanford University
Name and contact information for the trial sponsor {5b}	National Institute on Aging (program officer: Maggie Nellissery, Maggie.Nellissery@nih.org)
Role of sponsor {5c}	The sponsor has no role in the study design; collection, management, analysis, or interpretation of the data; writing of study reports; or decision to submit reports for publication

## Introduction

### Background and rationale {6a}

One in three women aged 60 years or older suffers from involuntary leakage of urine (urinary incontinence), with up to half of women with incontinence reporting leakage that occurs in the setting of strong or sudden urges to urinate (urgency incontinence) [1–3]. Among older women living in the community, urgency incontinence is associated with depression, social isolation, physical inactivity, functional decline, falls and fractures, and burden on caregivers [4–9]. In economic analyses, the estimated direct costs of urgency incontinence among US adults exceeded $80 billion in 2020, with a greater cost burden born by women than by men [10].

In older populations, urinary incontinence is often complicated by comorbid conditions that can exacerbate its impact and complicate management. One of the most common of these is cognitive impairment [11–13], including both dementia and mild cognitive impairment (MCI) that can become progressively more severe with time. As adults age, the combination of urinary incontinence and cognitive impairment can contribute synergistically to functional disability [14], increasing the likelihood that individuals will lose their ability to live independently, exhaust their informal caregivers, and require long-term care in older age [8, 15].

Currently, the most widely prescribed treatments for urgency-type incontinence in women of all ages are anticholinergic bladder medications that decrease involuntary contractions of the bladder. Unfortunately, anticholinergic medications block the action of acetylcholine, a key neurotransmitter involved in nerve cell communication and strongly implicated in memory function and dementia including Alzheimer disease (AD), and thus can precipitate short-term confusion in older adults. Observational studies have also reported higher rates of dementia diagnosed in older adults prescribed anticholinergic bladder drugs [16–19]. As a result, there is growing concern that older adults who initiate anticholinergic bladder therapy to improve their bladder control may risk developing even more serious functional compromise due to accelerated cognitive impairment [20].

To date, however, the evidence linking anticholinergic bladder therapy with cognitive impairment is derived primarily from observational data rather than prospective trials. The few studies directly assessing change in cognition in older patients from anticholinergic bladder therapy have been small or short or have had other methodologic limitations that may have affected their detection of cognitive effects [21–25]. As a result, many clinicians question whether reported associations with dementia from observational research represent a true, direct effect of these drugs or simply reflect confounding from greater underlying vulnerability to dementia among older, frail patients with incontinence [26–28]. In the absence of rigorous prospective evidence, anticholinergic bladder medications continue to be widely prescribed to older incontinent patients, including those with mild cognitive impairment or dementia [29–31].

An alternate pharmacologic treatment option for adults with urgency incontinence wishing to avoid anticholinergic bladder therapy is beta-3-adrenergic agonist medication (e.g., mirabegron and more recently vibegron). However, past research involving beta-3-adrenergic agonist therapy has not included a rigorous or comprehensive evaluation of cognitive function. Furthermore, past studies of beta-3-adrenergic agonist medication have included relatively few older adults with frequent incontinence to address some patients’ and clinicians’ concerns that it may not be as effective as anticholinergic therapy in controlling incontinence as the most severe manifestation of overactive bladder [32–34]. As a result, there is a need for high-quality clinical trial evidence of the effects of anticholinergic and beta-3-adrenergic agonist medications on both urinary incontinence and cognitive impairment, two of the most common (and frequently comorbid) chronic conditions causing older women to transition from living independently to needing long-term care.

Similarly, past trials have provided limited data to evaluate the effects of these medications on other important aging-related functional domains in older adults, including physical mobility and balance, psychological function, sleep, and bowel function. Without more evaluation of the effects of medication effects on these and other aspects of functioning and well-being, clinicians cannot make evidence-based decisions about whether to prescribe anticholinergic bladder medication to older adults with incontinence, alternately prescribe beta-3-adrenergic agonist medication to these patients, or instead refrain from prescribing medications entirely.

### Objectives {7}

The primary objective of the TRIUMPH trial is to compare the effect of anticholinergic bladder pharmacotherapy versus (a) beta-3 adrenergic agonist bladder pharmacotherapy and (b) no overactive bladder pharmacotherapy on overall (composite) cognitive function in older ambulatory women with urgency-predominant incontinence. The secondary objective of the trial is to compare the effects of anticholinergic bladder therapy versus beta-3 adrenergic agonist therapy and versus no bladder pharmacotherapy on domain-specific cognitive function; frequency, severity, and impact of participants’ urgency incontinence and other urgency-associated urinary symptoms; perceived sleep quality and daytime sleepiness; perceived and objectively assessed physical function and balance; psychological function including depression and anxiety symptoms; and bowel symptoms including constipation and bowel incontinence in this population.

## Methods: participants, interventions, and outcomes

### Trial design {8}

The TRIUMPH trial is a randomized, double-blinded, superiority trial using block randomization to assign participants in a 1:1:1 allocation to one of three parallel groups (anticholinergic, beta-3-adrenergic, or placebo bladder antispasmodic medication for 24 weeks), with participants in all treatment arms also receiving education about first-line behavioral self-management of incontinence, and designed to examine a primary endpoint of change in composite cognitive function 24 weeks after initiation of study treatments.

### Study setting {9}

The TRIUMPH trial is conducted by investigators and clinical research staff based at the University of California San Francisco (UCSF) and Stanford University, two academic medical research institutions based in northern California, USA. Study procedures are conducted in research clinics affiliated with UCSF or Stanford University or using online and videoconference platforms supported by these institutions. The majority of participants are expected to be residents of northern California, although participants may be recruited from other geographic areas if they can complete study procedures using remote platforms. Participants may include individuals who have received healthcare services from UCSF or Stanford University but are not limited to patients from these institutions.

### Eligibility criteria {10}

The eligibility criteria are designed to identify ambulatory older women with urgency-predominant urinary incontinence and no pre-existing dementia who are generalizable to the majority of older women in the community who are candidates for anticholinergic or beta-3-adrenergic agonist bladder pharmacotherapy. The eligibility criteria are designed to exclude individuals with a clinical history that poses a clinically significant safety issue for one of the study medications, but other exclusions have been kept to a minimum to promote generalizability. Men will not be enrolled due to the high prevalence of urinary symptoms attributable to prostatic hyperplasia in older men, which could warrant treatment with other medications such as alpha blockers that could have independent effects on the trial’s primary or secondary outcomes.

#### Inclusion criteria

To be eligible for randomization, participants must meet the following criteria:

- Aged 60 years or older at the time of enrollment
- Female sex at birth, without surgical or hormonal gender re-assignment therapy
- Able to walk to the bathroom and use the toilet without assistance
- Report urinary incontinence starting at least 3 months prior to screening
- Report that at least half of incontinence episodes occur with a sudden or strong sensation of urgency
- Report 2 or more urgency incontinence episodes over a 7-day period
- Willing to provide informed consent and adhere to the study procedures throughout the length of the study

#### Exclusion criteria

Candidates who meet any of the following criteria are excluded from randomization:

- Prior clinician diagnosis of dementia or a Montreal Cognitive Assessment (MoCA) score of 17 or lower on screening cognitive evaluation
- Current use of anticholinergic, beta-3-adrenergic agonist, or other bladder antispasmodic medications or use in the past 4 weeks
- Initiation, discontinuation, or dose change of dementia medications (such as donepezil, galantamine, memantine, rivastigmine) in the past month (4 weeks), although candidates on stable doses are eligible
- Initiation, discontinuation, or dose change of other drugs with strong anticholinergic effects (based on the Beers List [35]) in the past 4 weeks, although candidates on stable doses are eligible
- Initiation, discontinuation, or dose change of other drugs that can affect urinary frequency, including diuretics, in the past 4 weeks, although candidates on stable doses are eligible
- Current urinary tract infection (UTI) based on screening urinalysis and culture (but candidates can re-present for re-screening after undergoing treatment for UTI)
- History of allergy or sensitivity to either of the study medications or an ingredient in the placebo or study medication capsule
- Severe hepatic impairment (Child–Pugh score B or greater) or renal impairment (creatinine clearance < 30 mL/min) as a contraindication to both study medications
- Current bladder obstruction or urinary retention (defined by symptoms suggesting difficulty emptying the bladder in addition to postvoid residual urine volume greater than 150 cc by portable bladder ultrasound)
- Uncontrolled hypertension (based on measured systolic blood pressure greater than 180 or diastolic blood pressure greater than 110 mmHg) as a contraindication to beta-3-adrenergic therapy
- Self-reported history of gastric retention, uncontrolled narrow-angle glaucoma, myasthenia gravis, severe ulcerative colitis, or toxic megacolon as contraindications for anticholinergic bladder therapy
- Use of drugs with adverse interactions with one of the study medications in the past 4 weeks, including potent CYP3A4 inhibitors, hepatic enzyme metabolism inducers, narrow therapeutic index drugs metabolized by CYP2D6, or intention to start taking one of these medications during the study treatment period
- History of bladder surgery, invasive intra-vesical therapy, or bulk bladder injections in the past 12 weeks (more remote surgery will not be exclusionary) or intention to undergo one of these procedures in the study treatment period
- Use of other specialized incontinence therapy (electrostimulation, pelvic physiotherapy, formal behavioral therapy overseen by certified practitioners) in the past 12 weeks (more remote therapy will not be exclusionary) or intention to undergo one of these procedures in the study treatment period
- Inability to sign informed consent or complete questionnaires, interviews, or study testing in English
- Other conditions that would prevent the participant from completing study procedures, in the opinion of the investigators (e.g., uncontrolled psychosis)

### Who will take informed consent? {26a}

Clinical research coordinators under the supervision of the faculty investigators at UCSF or Stanford University will explain the study requirements to all potential trial participants before obtaining and documenting informed consent prior to or at the beginning of the screening visit. Consent may be documented on paper or using an online platform approved by the trial’s institutional review board (IRB) for documented consent.

### Additional consent provisions for the collection and use of participant data and biological specimens {26b}

Not applicable—the study team has no current plans for additional collection or use of data or biological specimens or for additional studies using the data collected in this trial.

### Interventions

#### Explanation for the choice of comparators {6b}

The TRIUMPH trial is designed to provide evidence of the potential beneficial and adverse effects of using the most common pharmacologic treatment strategy for urgency incontinence (anticholinergic bladder antispasmodic medication) versus (a) the main alternative pharmacologic treatment strategy (beta-3-adrenergic agonist bladder antispasmodic medication) or (b) no pharmacologic treatment for urgency incontinence (placebo medication). Since the primary goal of the trial is to address questions about the risk-to-benefit ratio of anticholinergic bladder medications in older women with incontinence, all trial hypotheses involve a comparison of anticholinergic bladder medication to another strategy (either beta-3-adrenergic agonist medication or placebo). No pre-specified comparisons of beta-3-adrenergic agonist medication to placebo are planned, as the study team does not have a strong a priori scientific reason to suspect that beta-3-adrenergic agonist treatment will have a differential effect on the primary cognitive outcome compared to placebo. Tolterodine tartrate and mirabegron have been chosen as the anticholinergic and beta-3-adrenergic agonist medications in this trial, respectively, because they represent the most widely prescribed medications in each of these categories in the USA as of the time of the trial design.

Since all pharmacologic therapy is considered by consensus guidelines to represent second-line treatment for urgency incontinence [36, 37], all trial arms also include short participant education on standard, evidence-based, behavioral self-management strategies for incontinence, consistent with first-line behavioral incontinence care, regardless of study medication allocation. Third-line treatments such as invasive bladder procedures are not examined, because these treatments are not recommended in routine clinical practice unless individuals first try and fail second-line pharmacologic treatments.

#### Intervention description {11a}

Randomized participants are instructed to take a daily dose of tolterodine tartrate, mirabegron, or placebo, identically encapsulated by a research compounding pharmacy:

- Tolterodine tartrate is a muscarinic receptor antagonist approved by the US Food and Drug Administration (FDA) for the treatment of urgency incontinence, urgency, and frequency symptoms associated with overactive bladder. After oral administration, tolterodine is metabolized in the liver, resulting in the formation of the 5-hydroxymethyl derivative, its pharmacologically active metabolite, which exerts its effects on muscarinic receptors found in the bladder detrusor muscle. For this trial, participants assigned to anticholinergic bladder therapy take tolterodine from 2 to 4 mg ER daily, based on participant-directed dose adjustment in the first 4 weeks.
- Mirabegron is a selective beta-3-adrenergic receptor agonist, also approved by the FDA for the treatment of symptoms of overactive bladder. Mirabegron relaxes the detrusor smooth muscle during the storage phase of the urinary bladder fill-void cycle by activation of beta-3 adrenoreceptors, to increase bladder capacity and decrease detrusor pressure. For this trial, participants assigned to beta-3-adrenergic agonist therapy are instructed to take mirabegron 25 to 50 mg ER, also with participant-directed dose adjustment in the first 4 weeks.
- Placebo therapy takes the form of a microcrystalline cellulose placebo encapsulated to appear identical to tolterodine and mirabegron by a compounding pharmacy.

To simulate medication dose adjustments for older patients in outpatient practice, study medications are initiated at low doses (i.e., tolterodine 2 mg ER, mirabegron 25 mg ER, or identically appearing placebo), but participants can increase medication dose without unblinding (to tolterodine 4 mg ER, mirabegron 50 mg ER, or identical placebo) based on their perception of treatment benefit and tolerability at 2 weeks. Participants may subsequently de-escalate medication dose based on an interim phone call at 3 weeks, before the 4-week study visit corresponding to the first outcomes assessment. After 4 weeks, the medication dose may be de-escalated if a participant develops a safety or tolerability issue, but no dose adjustment is scheduled, and dose increase is not permitted.

All participants also receive identical written information and brief teaching on evidence-based behavioral self-management strategies for incontinence, similar to the information provided during primary care visits. At the baseline visit, immediately prior to randomization, participants receive a pamphlet entitled “Staying Dry: A Practical Guide to Bladder Control” adapted from a workbook published under the same name [38], which provides evidence-based, patient-directed information about pelvic floor exercises, bladder training, and urge suppression techniques, and receives brief instruction from a trained study coordinator for practicing these techniques. At follow-up study visits, participants are asked about their practice of these techniques and their perceptions of the benefit of these techniques.

#### Criteria for discontinuing or modifying allocated interventions {11b}

Study medication may be discontinued on a case-by-case basis if deemed necessary to protect the safety of a participant or preserve the integrity of the study. Possible reasons for discontinuation of study medication include (1) development of persistent severe hypertension defined by a measured blood pressure greater than 180/110, (2) development of significant urinary retention or inability to empty the bladder, (3) apparent allergic reaction to study medication, (4) development of another clinically significant adverse event limiting a participant’s ability to safely continue to use study drug, (5) unusually disruptive behavior exhibited by a participant that endangers the safety or well-being of the participant or study staff, or (6) decision to terminate the study by the IRB, the sponsor, or other regulatory bodies. Participants may also decide to discontinue study medication or withdraw voluntarily from the study at any time and for any reason.

#### Strategies to improve adherence to interventions {11c}

Study coordinators review participant adherence to medications at each scheduled follow-up visit or telephone call and address barriers to adherence in the event that participants appear to have missed days of medication use. Adherence to study medication is assessed using study medication diaries in which participants are asked to record their medication use, as well as a tabulation of unused medication at the study end.

#### Relevant concomitant care permitted or prohibited during the trial {11d}

Participants are instructed to refrain from using other second- or third-line clinical treatments for incontinence during the study treatment or follow-up periods, including the following:

- Other anticholinergic bladder medications or other beta-3-adrenergic agonist bladder medications that are FDA approved for the treatment of urgency-associated urinary symptoms
- Surgical treatments for urinary incontinence or other invasive bladder therapies (e.g., botulinum injections into the bladder)
- Electrical or magnetic nerve stimulation therapy for urinary incontinence, including electroacupuncture; formal behavioral incontinence treatment programs administered by certified practitioners (e.g., pelvic floor rehabilitation therapy)

Participants are also asked to refrain from starting, stopping, or changing the dosage of several other types of medications that could affect their urination during the study treatment or follow-up periods, such as diuretic medications. However, the use of these medications is not exclusionary, as participants already using these medications prior to enrollment can continue to use them at steady doses during the study treatment and follow-up periods:

- Medications with strong anticholinergic properties (based on the Beers List) that could complicate the assessment of cognitive function during the study
- FDA-approved medications for dementia or cognitive impairment, such as anticholinesterase inhibitors, glutamase regulators, or aducanumab
- Loop diuretic medications or other medications with a strong effect on frequency or volume of urination

Participants are also permitted to use other self-directed behavioral management strategies or complementary management approaches for incontinence, such as the following:

- Self-directed pelvic floor muscle exercises (based on written or online information)
- Herbal medications, nutritional supplements, or homeopathic therapies
- Acupuncture that does not involve electrical stimulation

#### Provisions for post-trial care {30}

Participants are encouraged to seek evaluation and/or treatment from their usual sources of healthcare for any adverse health symptoms or problems that develop during the trial. If a participant recruited from UCSF is harmed because of study participation, the University of California will provide necessary medical treatment, with the costs of the treatment being billed to the participant or the participant’s insurer similar to other medical costs, or covered by the University of California depending on a number of factors. At Stanford University, if a participant develops medical complications from participating in this study, she will be assisted in obtaining appropriate medical treatment; in the event that the injury or illness is directly caused by study participation, reimbursement for all related costs of care first will be sought from the participant’s insurer, managed care plan, or other benefits program, and the participant will be responsible for any associated co-payments or deductibles as required by her insurance. The study institutions do not anticipate providing any other form of compensation for injury.

### Outcomes {12}

The primary outcome of change in composite cognitive function over 24 weeks of treatment is assessed using a standardized battery of neuropsychological tests selected for their sensitivity in evaluating relevant cognitive domains and feasibility of administration during either in-person or telehealth visits. Study coordinators undergo dedicated standardized training in administering these tests by a neuropsychology co-investigator, who also oversees the quality of these assessments. Specific tests include the following:

- The Rey Auditory Verbal Learning Test (AVLT) [39–41]
- The oral version of the Trail Making Test (OTMT) [42–45]
- The Digit Span Test from the Wechsler Adult Intelligence Scale [46]
- The Digit Symbol Substitution Test (DSST) [47, 48]

Baseline data on cognitive test performance in the full sample of randomized participants will provide the normative standard for cognitive test performance at both baseline and follow-up visits. For each cognitive test (or test component), the mean score from baseline administration of the test in the overall study population will be subtracted from each individual participant’s test score, and this difference will be divided by the standard deviation of test scores from the baseline trial population. After scores from individual cognitive tests are transformed to *Z* scores as a common metric using this approach, the average *Z* score from all available tests completed by each participant at each visit will be calculated to provide a composite *Z* score for the participant at that visit. The primary outcome will be described as the modeled mean change from baseline in this composite cognitive *Z* score at 4, 12, and 24 weeks, averaged over these time points.

Secondary domain-specific cognitive outcomes are defined by model-based mean changes from baseline in scores on each of the above individual cognitive tests (AVLT total learning, AVLT delayed free recall, OTMT part A, OTMT part B, Digit Span Test backward component, and DSST), also assessed over 24 weeks of treatment (averaged over 4, 12, and 24 weeks). Additional secondary cognitive outcomes are defined by modeled mean persistent difference in composite cognitive function score as well as scores on each of the above domain-specific cognitive tests at 12 weeks after the end of treatment (36 weeks after randomization).

Secondary urinary outcomes are defined by the modeled mean change from baseline in the following voiding diary measures and questionnaire scores over 24 weeks of treatment (averaged over 4, 12, and 24 weeks), as well as at 12 weeks after the end of treatment (36 weeks):

- Frequency of urgency-type incontinence (episodes/week)
- Frequency of any-type incontinence (episodes/week)
- Resolution of urinary incontinence (dichotomous outcome)
- Overactive Bladder Questionnaire Short-Form (OAB-Q SF) Symptom Bother domain score [49]
- Overactive Bladder Questionnaire Short-Form (OAB-Q SF) Health-Related Quality of Life domain score [49]

Secondary sleep-related outcomes are defined by the modeled mean change from baseline in scores in the following questionnaire measures over 24 weeks of treatment (averaged over 4, 12, and 24 weeks), as well as at 12 weeks after the end of treatment (36 weeks):

- Pittsburgh Sleep Quality Index (PSQI) global sleep quality score [50, 51]
- Epworth Sleepiness Scale (ESS) score [52]

Secondary physical function and balance outcomes will be defined by the modeled mean change from baseline in the following questionnaire and physical performance assessments over 24 weeks of treatment (averaged over 4, 12, and 24 weeks), as well as at 12 weeks after the end of treatment (36 weeks):

- PROMIS 10B Adult Physical Function Scale [53]
- Activities Balance Confidence Scale (ABC-S) [54, 55]
- Short Physical Performance Battery (SPPB) [56, 57]
- One-legged standing balance time [58, 59]
- Thirty-second chair stand test [60]

Secondary psychological function outcomes will be defined by the modeled mean change from baseline in scores in the following questionnaire measures over 24 weeks of treatment (averaged over 4, 12, and 24 weeks), as well as at 12 weeks after the end of treatment (36 weeks):

- Geriatric Depression Scale-15 (GDS-15) [61]
- Generalized Anxiety Depression-7 (GAD-7) [14] scale

Secondary bowel function outcomes will be defined by the modeled mean change from baseline in scores in the following questionnaire measures over 24 weeks of treatment (averaged over 4, 12, and 24 weeks), as well as at 12 weeks after the end of treatment (36 weeks):

- PROMIS Gastrointestinal Symptoms-Constipation scale [62, 63]
- PROMIS Bowel Incontinence scale [62, 63]

### Participant timeline {13}

**Fig. 1:**
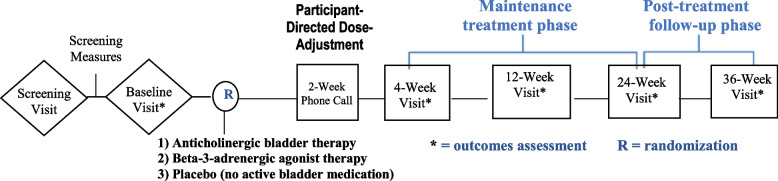
Study design

Participants complete the screening measures before randomization at a baseline visit that must take place within 2 months (60 days) of screening. Initiation of interventions begins immediately after randomization, after which participants are scheduled for follow-up at 2 weeks for consideration of study medication dose titration. Follow-up visits to collect data for the trial’s primary and secondary on-treatment outcomes take place at 4 weeks, 12 weeks, and 24 weeks. An additional 36-week visit is scheduled to collect data on the potential persistence of treatment effects 12 weeks after participants have discontinued study treatment (Fig. 1).

### Sample size {14}

A sample size of 270 participants, randomized in equal proportions to the three arms, has been chosen to provide > 80% power in 2-sided tests with an experiment-wise type 1 error rate of 5%, corrected for two comparisons (anticholinergic therapy to beta-3-adrenergic agonist therapy; anticholinergic therapy to placebo) [48] to detect an average treatment effect of 0.25 standard deviation’s difference in the composite cognitive function outcome over 6 months, assuming an intraclass correlation of 0.75 among repeated cognitive outcomes, and cumulative loss to follow-up level of 15% by 6 months of treatment.

### Recruitment {15}

The TRIUMPH trial team is using a multi-pronged recruitment approach to recruit older ambulatory incontinent women residing in multiple communities in northern California. Study candidates are identified through community-based recruitment strategies, such as the mailing of recruitment letters to households in northern California using commercial mailing services; posting of recruitment fliers in local senior centers, community organizations, or businesses; recruitment advertisements placed in regional newspapers; and/or online social media-based recruitment. Recruitment may also include mailings to current and past female patients aged 60 years or older who have received healthcare services from UCSF Health and Stanford Medical Center as the healthcare delivery systems associated with the study institutions, as well as recruitment from a database of past participants from other UCSF or Stanford-based studies of urinary symptoms or cognitive decline.

### Assignment of interventions: allocation

#### Sequence generation {16a}

Eligible participants are randomized in a fixed 1:1:1 ratio to each of the study arms, with stratification by study site (UCSF versus Stanford University), using randomly permuted blocks of sizes of 4 and 6. The randomization scheme has been generated by computer by a statistician independently contracted through the UCSF Clinical and Translational Science Institute.

#### Concealment mechanism {16b}

An independent statistician has been contracted to prepare the allocation sequence, provide the sequence to the research pharmacy responsible for dispensing study medication, and upload the sequence into the electronic study data system, where it is concealed from the trial investigators, coordinators, and statistical programmers.

#### Implementation {16c}

At baseline, after screening data have been reviewed and eligibility has been confirmed, a blinded coordinator enters the date into the electronic data system to retrieve the randomization number, which enforces temporally sequential randomization assignments. The coordinator submits the randomization number to the contracting research pharmacy, who identifies and dispenses the study medication lot associated with the randomization number.

### Assignment of interventions: blinding

#### Who will be blinded {17a}

Consistent with a double-blinded design, all participants, investigators, and study staff who have a role in promoting adherence or assessing or documenting outcomes are blinded to the treatment assignment. During the main part of the trial, only the statistician responsible for generating the randomization scheme and the compounding pharmacy have access to the treatment assignment information. Study-wide unblinding will take place when data from all trial data forms and measurements have been entered into the study database for all participants, data cleaning for the trial has been completed, the data have been locked, and the principal investigator has declared the study dataset to be complete.

#### Procedure for unblinding if needed {17b}

Unblinding prior to the study-wide unblinding date will be undertaken only in exceptional circumstances in which knowledge of a participant’s actual intervention assignment is essential to protect the safety of a participant or another individual. Examples include the occurrence of a serious adverse event for which knowledge of the participant’s assignment could affect her immediate care or consumption of study drug in excessive or toxic quantity by the participant or by another party. The decision to unblind will be made on a case-by-case basis by the principal investigator, in consultation with the Steering Committee or DSMB as needed, and knowledge of treatment assignment will be restricted to the smallest number of people possible. If the decision to unblind a participant is made, an analyst who is not otherwise involved in collecting, cleaning, or interpreting trial data will consult the list of randomization numbers to access treatment assignment information for the participant’s randomization number, documenting the date and reason for unblinding. Treatment assignment will be revealed for the relevant individual participant only, so that unblinding of one participant does not compromise the blinding of other participants.

### Data collection and management

#### Plans for assessment and collection of outcomes {18a}

Cognitive function outcomes will be assessed using a battery of neuropsychological tests (see the “Outcomes {12}” section) administered by clinical research coordinators who have completed dedicated training and certification by a neuropsychiatry or neuropsychologist investigator, who will also oversee the quality of these assessments. Whenever possible, repeated cognitive measures in the same participant will be administered by the same evaluator and use the same mode of administration (in person versus by video visit) across all study time points. Additionally, a sample of session recordings will be observed by the neuropsychologist co-investigator to ensure standardized administration across coordinators.

Frequency and type of incontinence will be assessed using a standardized, 7-day voiding diary. At baseline and outcome follow-up visits, participants will receive a blank diary and instructions on using it to record each time they leak urine as well as indicate whether the leakage is associated with a sudden or strong urge to urinate. Diary data will be abstracted by blinded analysts who have undergone study-specific training and supervised practice in reviewing and abstracting diary data to calculate the frequency of incontinence associated with urgency.

Structured self-report questionnaires will be used to assess other urinary, sleep-related, perceived physical function, psychological, and bowel function outcomes (see the “Outcomes {12}” section). Participants may complete the questionnaires electronically or alternately complete them on paper and then return them to blinded study coordinators for electronic data entry.

Physical performance and balance testing will be performed by clinical research coordinators who have completed study-specific training in these measures. Testing will be performed in person or through video-based visits, following procedures outlined in the study manual of operations, but the same mode of assessment will be preferentially used at baseline and follow-up assessments.

#### Plans to promote participant retention and complete follow-up {18b}

To promote retention and follow-up, participants are offered multiple methods of completing follow-up visits and measures. Cognitive and physical function assessments can be completed in person in a study clinic or via videoconference-based study visits. Study questionnaires can be completed on paper or online, either in a study clinic or at home. Participants receive reimbursement for their time and trouble completing study follow-up visits and measures. Participants are encouraged to complete all study outcome and safety monitoring measures even if they discontinue study interventions early.

#### Data management {19}

Study data are electronically entered, managed, and edited by clinical coordinators or other study staff using the Medrio web-based Electronic Data Capture software for Clinical Research, a secure, 21 Code of Federal Regulations Part 11 compliant web-based application designed for research data entry and management. The Medrio system automatically generates queries for missing data or out-of-range values based on initial programming by analysts at the UCSF Data Coordinating Center. As described in the Data and Safety Monitoring Plan (DSMP), the UCSF Data Coordinating Center under the supervision of the principal investigator provides a secondary layer of oversight for data accuracy and completeness by tracking data queries posted in the electronic data capture system, prompting site-specific study personnel to address data queries for missing or out-of-range data in a timely manner, and generating study-wide reports for reviews of missing data or data outliers. Overall data completeness and quality are periodically assessed by the UCSF Data Coordinating Center using measures such as number of missing data forms and number of outstanding data queries flagged within this system.

#### Confidentiality {27}

All study-related information is stored securely at the study site or using secure electronic platforms. All paper-based participant information is stored in locked file cabinets in research facilities with limited access. All data collection, process, and administrative forms are identified by a coded participant ID (identification) number only to maintain participant confidentiality. All records that contain names or other personal identifiers, such as informed consent forms, are stored separately from study records identified by code number. All local databases are secured with password-protected access systems with dual-factor authentication. Forms, lists, logbooks, appointment books, and any other listings that link participant ID numbers to other identifying information are stored in a separate, locked file in research facilities with limited access. Participants’ study information will not be released outside of the study without the written permission of the participant, except as necessary for monitoring by the sponsor and/or its contractors or by government or regulatory authorities.

#### Plans for collection, laboratory evaluation, and storage of biological specimens for genetic or molecular analysis in this trial/future use {33}

Not applicable—the TRIUMPH trial does not include any plans for collection, laboratory evaluation, or storage of biological specimens.

## Statistical methods

### Statistical methods for primary and secondary outcomes {20a}

To examine the primary outcome, medication effects on cognitive function will be estimated using a linear mixed model (LMM) for repeated changes from the baseline of the composite cognitive function outcome, averaged at 4, 12, and 24 weeks, adjusted for baseline value. In addition to a fixed effect for study location, the LMM will include terms for time (as a categorical variable) and time-by-treatment interactions. Treatment effects will be captured by the time-by-treatment interactions and their 95% confidence intervals, focusing on the comparisons of the anticholinergic arm with each of the two other arms.

The primary hypothesis will be tested using the average of the time-by-treatment interactions at 4, 12, and 24 weeks. Persistence of treatment effects will be evaluated by examining the time-by-treatment interaction at 36 weeks, and second by contrasting the 36-week interaction with the average of the 4-, 12-, and 24-week interactions, which will capture attenuation of the on-treatment effect after an additional 12 weeks. In models involving the average of the effects at 4, 12, and 24 weeks, we will check for heterogeneity and trend across those visits. If evidence of heterogeneity is detected, we will consider examining time-by-treatment interactions at each time point (4, 12, and 24 weeks) separately.

The primary analyses will be by intention to treat among participants with at least one post-baseline follow-up assessment, according to treatment group assignment, and without regard to adherence, avoiding selection bias from informative discontinuation of treatment. However, secondary per-protocol analyses omitting those outcomes will also be performed, since the inclusion of post-discontinuation outcomes may attenuate treatment effect estimates if treatment effects are primarily acute.

For secondary outcomes, treatment effects will also be estimated using similar LMMs based on change from baseline in measures at 4, 12, 24, and 36 weeks, adjusted for baseline values of outcomes. Once again, treatment effects will be captured by the time-by-treatment interactions and their 95% confidence intervals, focusing on the comparisons of the anticholinergic arm with each of the two other arms. Analyses will again be by intention to treat, according to treatment group assignment, and without regard to adherence; per-protocol analyses, as described above for the primary outcome, will also be performed for secondary outcomes. The study team will again examine the average of the time-by-treatment interactions at 4, 12, and 24 weeks, while checking for heterogeneity and trend across those visits, and then contrast the 36-week interactions with the average of the 4-, 12-, and 24-week interactions. No penalization for multiple comparisons will be made, but the results will be presented as secondary outcome analyses.

### Interim analyses {21b}

This trial focuses on medications that are already in widespread use in the community for urgent incontinence and administers these medications according to their already FDA-approved indications and designated populations. The trial is designed to follow participants for limited time periods (24 weeks of medication treatment, as well as 12 weeks after discontinuation of treatment), using self-report measures and other non-invasive data collection procedures that impose only a modest burden on participants. As a result, the investigators do not believe there is a compelling scientific or ethical rationale to stop the trial earlier than planned even if differences in the trial’s cognitive, urinary, or functional outcomes begin to emerge earlier than expected. Early termination of the trial based on preliminary data trends could increase the risk of spurious conclusions and endanger the credibility of the findings in the broader clinical community, especially given that many clinicians may have strong preferences or biases related to these medications. Therefore, no interim treatment analyses for the trial’s cognitive, urinary, or other functional outcomes are planned in advance.

### Methods for additional analyses (e.g., subgroup analyses) {20b}

Intervention effects will be analyzed in subgroups in multivariate analyses based on a limited set of clinicopathologic factors that are hypothesized to have the potential to influence intervention effects. For all outcomes, the investigators will explore whether participant age (< 75 vs. ≥ 75 years) modifies the effects of treatment, unless this is precluded by a minimal subgroup sample size. Other pre-specified subgroup analyses include the following:

- Baseline cognitive function status (MoCA score < 26 vs. ≥ 26–30) for cognitive function outcomes
- Baseline frequency of incontinence (daily vs. less than daily incontinence), for urinary symptom outcomes
- Baseline global physical performance (SPPB score < 10 vs. ≥ 10), for physical function or performance outcomes
- Baseline poor sleep quality (PSQI score > 5 vs. ≤ 5), for sleep-related outcomes
- Baseline depression (GDS-15 ≥ 9 vs. < 9) or anxiety (GAD-7 ≥ 10 vs. < 10) symptom score, for depression or anxiety outcomes, respectively

Subgroup-specific effects will be presented only if the interaction between treatment and subgroup is statistically significant at *p* < 0.05.

### Methods in analysis to handle protocol non-adherence and any statistical methods to handle missing data {20c}

Primary analyses will be by intention to treat among participants with at least one post-baseline follow-up assessment, according to treatment group assignment, and without regard to adherence, avoiding selection bias from informative discontinuation of treatment. However, secondary, per-protocol analyses omitting those outcomes will also be performed. If rates of participant dropout or missing data differ between the groups, multiple imputation of missing outcomes may be performed under plausible informative missingness assumptions.

### Plans to give access to the full protocol, participant-level data, and statistical code {31c}

Starting no later than 6 months following the publication of the main trial results (including online publication), the investigators will make publicly available de-identified individual participant data that underlie the results reported in the publication. This will include data about the baseline characteristics of the study participants and any primary or secondary study outcomes presented in the publication. To gain access, data requestors will be asked to sign a data access agreement.

No later than 5 years of the conclusion of data collection (i.e., defined by the last participant contact), the investigators plan to deposit a complete trial dataset in a public data repository platform such as the UCSF integrated data repository and include the study protocol and a data dictionary with trial data.

### Oversight and monitoring

#### Composition of the coordinating center and trial steering committee {5d}

The primary governing body of the study is the TRIUMPH Steering Committee, composed of the principal investigator, co-investigators, and faculty statistician. The Steering Committee directs all aspects of the study, including protocol design, development of the operations manual, selection of data collection instruments, monitoring of study progress and quality, and resolution of issues that arise during follow-up. The Steering Committee serves as a Publications and Ancillary Studies Committee to review and approve the proposals for ancillary studies, analyses, and reports or presentations. Independent monitoring of study safety and quality is also provided by a DSMB, consisting of members appointed by the NIA as the study sponsor, who are independent of the institution and investigators involved in the study and have no financial, scientific, or other conflicts of interest with the trial. The data management team will develop a data collection system and produce data quality reports to be reviewed by the DSMB as well as Steering Committee members at staff meetings.

#### Composition of the data monitoring committee, its role, and reporting structure {21a}

The conduct of the study and safety of participants is evaluated by an independent data and safety monitoring board (DSMB) composed of a minimum of three members. All members of the DSMB are independent of the investigators and staff participating in the study and have no financial ties to the study outcome. Collectively, the DSMB members have experience in general medicine or geriatrics, urology, clinical trial methodology, and biostatistics necessary to provide appropriate oversight for the proposed research. No DSMB members will participate in the study as an investigator or be involved in any way in the conduct of the study.

Prior to the initiation of the trial, the DSMB reviewed and approved the study design and plans for recruitment, adherence, interventions, data quality, and safety monitoring. Approximately twice a year after recruitment begins, the DSMB will evaluate the adequacy and timeliness of participant recruitment, adherence to the protocol, and the potential of the study to meet the stated goals; the quality and integrity of the data, including the adequacy of data management and data security procedures; participant safety including trends in adverse events and relationship to the study procedures; and factors external to the study when these may have an impact on the safety of the participants or the ethical conduct of the study. The DSMB will make recommendations, if necessary, to the investigators and the NIA on the continuation, termination, or other modifications of the study protocol.

#### Adverse event reporting and harms {22}

To monitor participant safety, clinical research coordinators assess for adverse events (AEs) at each scheduled follow-up telephone and in-person contact following randomization until the 36-week visit, starting with the 2-week telephone call. Additionally, participants are given telephone numbers to call the study staff in between scheduled visits or calls to report any significant health changes. Coordinators will encourage participants to volunteer information by asking the standardized, open-ended question, “Have there been any changes in your health since your last visit?”.

The TRIUMPH trial uses the NIA’s definition of an AE as any untoward or unfavorable medical occurrence in a study participant, including any abnormal sign (e.g., abnormal physical exam or laboratory finding), symptom, or disease, temporally associated with participants’ involvement in the research, whether or not considered related to participation in the research. In general, medical conditions or diseases present before starting study interventions are considered adverse events only if they worsen after starting the interventions. For study purposes, a serious adverse event (SAE) is any AE that (a) results in death, is life-threatening, or places the participant at immediate risk of death from the event as it occurred; (b) requires or prolongs hospitalization; (c) causes persistent or significant disability or incapacity, or (d) results in congenital anomalies or birth defects. Additionally, any other important medical event may be considered an SAE if it is judged by the investigators to jeopardize the safety of a participant or to require medical or surgical intervention to prevent one of the above outcomes listed in this SAE definition. An unanticipated problem (UP) is defined by NIA Adverse Event guidelines as any incident, experience, or outcome that meets all of the following criteria: (1) is unexpected, in terms of nature, severity, or frequency, given the research procedures described in the protocol and the characteristics of the study population; (2) is related or possibly related to participation in the research; and (3) suggests that the research places participants or others at a greater risk of harm (including physical, psychological, economic, or social harm) than was previously known or recognized.

Adverse events and SAEs will be categorized by organ system class using the Medical Dictionary for Regulatory Activities (MedDRA), severity/toxicity using the 5-point Common Terminology Criteria for Adverse Events (CTCAE) scale, likelihood of relationship to study interventions (unrelated, unlikely related, possibly related, probably related, or definitely related), and expectedness (expected, unexpected). SAEs and UPs will be reported to the IRB, the DSMB, and the NIA within the time frames specified by these entities, depending on whether events (a) are possibly related to study participation, (b) are unexpected, and/or (c) result in death or are life-threatening.

#### Frequency and plans for auditing trial conduct {23}

The TRIUMPH investigators and the NIA as the study funder have no current plans for external auditing of trial conduct.

#### Plans for communicating important protocol amendments to relevant parties (e.g., trial participants, ethical committees) {25}

The study protocol, informed consent document, data and safety monitoring plan, and data collection forms have been reviewed and approved by the study IRB prior to the implementation of study procedures. Any subsequent modifications to these documents will also be reviewed and approved by the study IRB prior to administration in the study. Additionally, any modifications to the study protocol and data and safety monitoring plan will be approved by the DSMB and the NIA prior to implementation.

#### Dissemination plans {31a}

Upon completion of data collection, summary results information for the trial will be submitted to ClinicalTrials.gov for public posting. This will include information about the number of participants starting and completing the study, estimated effects of the trial interventions on the primary and secondary outcomes, and important adverse events experienced by study participants. Results information will be submitted not later than 1 year after the trial’s primary completion date, which will be defined as the completion of all data collection activities for the primary outcome measure.

Additionally, the investigators will submit the results of this trial for publication in one or more peer-reviewed medical journals after data collection is complete, no later than 1 year after the trial’s completion date. Trial results may be presented in a single publication or divided into multiple publications, based on the judgment of the Steering Committee. In the event that the trial cannot be completed (due to early termination of the trial for safety or other reason raised by the trial’s DSMB, IRB, or other oversight body), interim results may still be submitted for publication if they are deemed worthy of increasing generalizable knowledge in the field. The investigators also intend to disseminate the results to the wider scientific and clinical community through a presentation at scientific and professional medical meetings.

## Discussion

The major challenge posed by the TRIUMPH trial design and procedures is the recruitment and retention of a generalizable population of older incontinent women who are similar to older female patients considering the use of anticholinergic and beta-3-adrenegic agonist medications in the community. Even though urgency incontinence disproportionately affects women who are older, frailer, and at risk for cognitive and physical functional decline, these individuals have been relatively under-represented in past studies of medications for urgency-associated urinary symptoms. Such individuals may have higher underlying rates of adverse events, both related or not related to study medications, which may increase the burden of complexity of safety monitoring. However, the lack of rigorous data on the multisystem effects of bladder medications in this population makes it important to attempt to enroll and engage this population.

## Trial status

This manuscript reflects TRIUMPH protocol version 1.5, created on December 8, 2022, and approved by the UCSF IRB, the NIA, and the TRIUMPH study DSMB. Recruitment began on September 19, 2022, and is anticipated to be completed by March 2026.

## Data Availability

The final TRIUMPH trial dataset will be stored and maintained at the UCSF Data Coordinating Center, where it will be accessible to all TRIUMPH investigators for quality monitoring. Other access to the study data will be available through a data request process overseen by a TRIUMPH Steering Committee composed of the principal investigator (PI), co-investigators (Co-Is), and statisticians. Data requestors seeking to use trial data to generate new publications or presentations will be asked to submit a publication/presentation proposal that will be reviewed by the members of the trial Steering Committee.

## Abbreviations

ABC-S: Activities Balance Confidence Scale
AD: Alzheimer disease
AVLT: Rey Auditory Verbal Learning Test
CTCAE: Common Terminology Criteria for Adverse Events
DSMB: Data and safety monitoring board
DSST: Digit Symbol Substitution Test
ER: Extended release
FDA: Food and Drug Administration
GAD-7: Generalized Anxiety Depression-7
GDS-15: Geriatric Depression Scale-15
IRB: Institutional review board
LMM: Linear mixed model
MedDRA: Medical Dictionary for Regulatory Activities
MCI: Mild cognitive impairment
MoCA: Montreal Cognitive Assessment
NIA: National Institute on Aging
OAB-Q SF: Overactive Bladder Questionnaire Short-Form
OTMT: Oral Trail Making Test
SPPB: Short Physical Performance Battery
TRIUMPH: Treating Incontinence for Underlying Mental and Physical Health
UCSF: University of California San Francisco
